# Port-Site Metastasis of Undiagnosed Pancreatic Adenocarcinoma After Laparoscopic Radical Prostatectomy: Case Report and Literature Review

**DOI:** 10.1089/cren.2018.0046

**Published:** 2018-09-18

**Authors:** Gotardo Zini Pinho, Gustavo Ruschi Bechara, Samira Pereira das Posses, Carla Regina Santos De Carli, Marcio Maia Lamy de Miranda

**Affiliations:** ^1^ Department of Urology, Federal University of Espírito Santo (UFES), Hospital Universitário Cassiano Antônio de Moraes–HUCAM, Vitória, Brazil.; ^2^ Department of Pathology, Federal University of Espírito Santo (UFES), Hospital Universitário Cassiano Antônio de Moraes–HUCAM, Vitória, Brazil.

**Keywords:** prostate cancer, laparoscopic radical prostatectomy, port-site metastasis, review

## Abstract

***Background:*** Laparoscopic port-site metastases remain rare for urologic tumors, despite the increasing use of laparoscopic techniques on the approach of urologic malignancy. Herein, we report a case of port-site mass after laparoscopic radical prostatectomy whose immunohistochemistry demonstrated metastasis from a pancreatic lesion.

***Case Presentation:*** A 62-year-old man presented to our ambulatory clinic with an elevated prostate-specific antigen (PSA) of 7.7 ng/mL. Transrectal biopsies revealed prostate cancer Gleason 6 (3 + 3) on the right side. He was subjected to a transperitoneal laparoscopic radical prostatectomy at our institution. The PSA on postoperative week 6 was 0.04 ng/mL. Three months after the surgery, he comes back to the emergency department complaining of an abdominal pain especially on the right flank. Our examination of the abdomen revealed a small palpable mass at the right upper port-site scar. Computed tomography of the abdomen and pelvis, with contrast, revealed a hypodense nodular lesion located on the abdominal wall near the upper port site and adjacent to the pancreatic tail. An excisional biopsy of the lesion confirmed the presence of metastatic adenocarcinoma. Immunohistochemistry demonstrated metastasis from a pancreatic lesion.

***Conclusion:*** Port-site mass after laparoscopic radical prostatectomy is uncommon especially in quite different tumors like this one with Gleason score 6 (3 + 3). Generally, port-site recurrences after a urologic laparoscopic surgery are uncommon and are not associated with diffused peritoneal carcinomatosis. Therefore, in this situation, another tumor site should be investigated as the primary source.

## Background

Radical prostatectomy is considered as a first-line treatment for men who were diagnosed with localized prostate cancer with a life expectancy greater than 10 years, and there are different treatment options, such as open radical prostatectomy, laparoscopic approach, or robot-assisted laparoscopic approach.^1^

Laparoscopic radical prostatectomy is a widely accepted technique for the treatment of localized prostate cancer, with oncological outcomes equivalent to open surgery.^1^

Despite the increasing use of laparoscopic techniques on the approach of urologic malignancy, laparoscopic port-site metastases and diffused peritoneal carcinomatosis remain rare for urologic tumors. When it occurs, another tumor site should be investigated.^2^

This article will review contemporary approaches to the management of port site mass after a urologic laparoscopic surgery in an attempt to identify the best practice for this unusual complication.

## Case Presentation

**FIG. 1.**
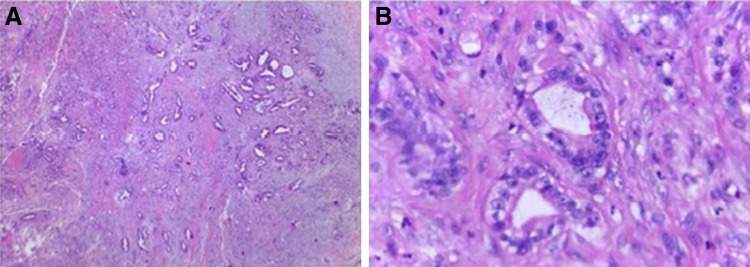
**(A)** Adenocarcinoma of the prostate Gleason score 3 + 3 = 6, grade group 1 (ISUP/WHO, 2016). **(B)** Higher magnification of Figure 1A.

**FIG. 2.**
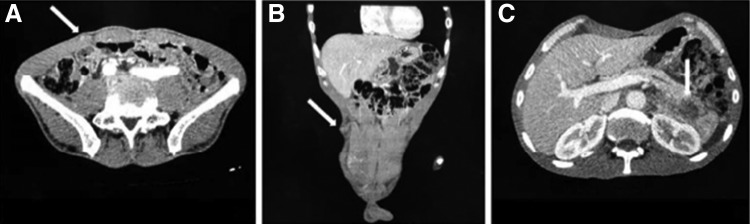
**(A, B)** Axial CT scan demonstrating a hypodense nodular lesion with barely defined contours located on the abdominal wall near the upper port site (*arrow*). **(C)** CT scan demonstrates a heterogeneous mass adjacent to the pancreatic tail (*arrow*).

**FIG. 3.**
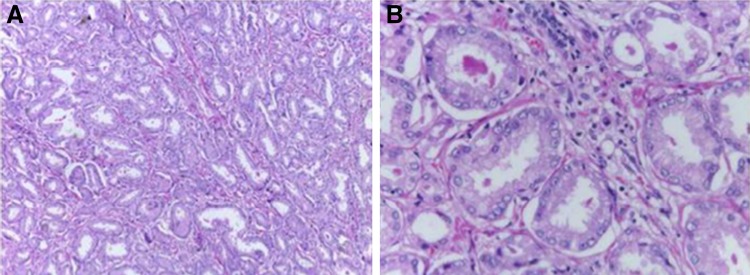
**(A)** Adenocarcinoma of acinar pattern infiltrating conjunctive tissue, compatible with metastatic adenocarcinoma. **(B)** Figure 3A amplified.

A 62-year-old man presented to our ambulatory clinic with an elevated prostate-specific antigen (PSA) of 7.7 ng/mL. The digital rectal examination revealed no changes. Transrectal biopsies were performed, revealing prostate cancer Gleason 6 (3 + 3) on the right side (apex). He was subjected to a transperitoneal laparoscopic radical prostatectomy at our institution (Federal University of Espírito Santo—HUCAM/UFES) in February/2017. The specimen was removed with a glove entrapment bag, and the port-site fascia was closed at the end of the surgery. Histopathological analysis confirmed prostate cancer pT2aNxMx, Gleason 6 (3 + 3) (Fig. 1). Urethral Surgical margin was positive and vesical margin was negative. The PSA, on postoperative week 6 was 0.04 ng/mL. Three months after the surgery, he comes back to the emergency department complaining of an abdominal pain especially on the right flank. Our examination of the abdomen revealed a small palpable mass at the right upper port-site scar. Computed tomography of the abdomen and pelvis, with contrast, revealed a hypodense nodular lesion with barely defined contours located on the abdominal wall near the upper port site and adjacent to the pancreatic tail measuring 1.7 and 4.1 cm, respectively (Fig. 2). The patient was subjected to diagnostic laparoscopy with pancreatic nodule biopsy followed by an excisional biopsy of the subcutaneous lesion, which showed pancreatic adenocarcinoma and presence of metastatic adenocarcinoma, respectively (Fig. 3). The material was sent to immunohistochemistry and a metastasis from pancreatic lesion was confirmed. An MRI of the pelvis and a bone scan did not reveal any changes. Currently, the patient is in a quarterly follow-up and did not present biochemical recurrence at this time.

## Discussion

Dobronte et al. reported the first case of port-site metastasis after a laparoscopic surgery.^3^ The incidence of the seeding tumor and port-site recurrence in this report published data ranging from 0.6% to 21%. It was suggested by authors that the incidence of port-site metastases after laparoscopic procedure was similar to open surgery. However, few cases have been reported in the literature.^4^

The first report of port-site metastasis involving a urologic procedure was described in 1994 after a lymphadenectomy for transitional cell carcinoma of the bladder. Castillo and Vitagliano published a brief review of the literature, which covered 17 studies, a total of 31 cases of port-site metastasis or secondary seeding tumor to laparoscopic urologic surgery in the past 20 years.^4^

The first case of a conventional prostate cancer with port-site metastases after laparoscopic radical prostatectomy was reported by Savage et al.^5^ This case revealed, besides metastasis on the port site, a hydroureteronephrosis and a left obturator lymph node and it was treated with androgen deprivation therapy demonstrating good response. Another cutaneous metastasis was reported by Bangma et al.^6^ The authors reported that spillage of tumor cells might have occurred during the dissection of a hard necrotic mass around the left obturator nerve.

Although some cases of port-site mass after laparoscopic surgery with unknown primary tumor have been described in the literature, those data are still not enough. Simonelli et al. reported a rare case of intraperitoneal mesh prosthesis metastasis from pancreatic cancer, after a laparoscopic hernia repairing.^7^

For port-site metastasis to occur, a lesion cell should loose from the primary tumor, adhere on another organ, and grow. Cell spillage can occur from inadvertent trauma due to instruments or other surgical instruments contaminating to an unprotected wound and it is also facilitated by poor immune status, advanced tumor stages, and the presence of ascites. The effect of gas turbulence in long laparoscopic procedures and the embolization or hematogenous dissemination are all possible mechanisms.^7^

Pancreatic metastases after laparoscopic radical prostatectomy are extremely rare. To the best of our knowledge, there are few studies of abdominal wall distant metastasis in the literature.

## Conclusion

Port-site metastasis after laparoscopic radical prostatectomy is uncommon especially in quite different tumors like this one with Gleason score 6 (3 + 3) and in this situation, another tumor site should be investigated as the primary source.

We report a port-site metastasis with diffused peritoneal carcinomatosis due to an unsuspected pancreatic adenocarcinoma diagnosed by an abdominal computed tomography, after a laparoscopic radical prostatectomy.

As more laparoscopic procedures are performed worldwide for oncological staging, similar cases might be published to complete our understanding of this rare pathology.

## Abbreviations Used

CT: computed tomography
MRI: magnetic resonance imaging
PSA: prostate-specific antigen

## References

[1] Heidenreich A, Bellmunt J, Bolla M, et al. EAU Guidelines on prostate cancer. Part 1: Screening, diagnosis, and treatment of clinically localised disease. Eur Urol 2011;59:61–71 21056534 10.1016/j.eururo.2010.10.039

[2] Reymond MA, Schneider C, Kastl S, Hohenberger W, Köckerling F The pathogenesis of port-site recurrences. J Gastrointest Surg 1998;2:406–414 9843599 10.1016/s1091-255x(98)80030-9

[3] Dobronte Z, Wittmann T, Karacsony G Rapid development of malignant metastases in the abdominal wall after laparoscopy. Endoscopy 1978;10:127–130 149005 10.1055/s-0028-1098280

[4] Castillo OA, Vitagliano G Port site metastasis and tumor seeding in oncologic laparoscopic urology. Urology 2008;71:372–378 18342166 10.1016/j.urology.2007.10.064

[5] Savage SJ, Wingo MS, Hooper HB, Smith MT, Keane TE Pathologically confirmed port site metastasis after laparoscopic radical prostatectomy: case report and literature review. Urology 2007;70:1222.e9–11 10.1016/j.urology.2007.09.004 18158058

[6] Bangma CH, Kirkels WJ, Chadha S, et al. Cutaneous metastasis following laparoscopic pelvic lymphadenectomy for prostatic carcinoma. J Urol 1995;153:1635–1636 7714993

[7] Simonelli V, Boven C, Loi P, El Nakadi I, Closset J Intraperitoneal mesh prosthesis metastasis from pancreatic cancer, after laparoscopic hernia repair. Acta Chir Belg 2016;116:51–53 27385143 10.1080/00015458.2016.1139831

